# Close Links between Cold Shock Proteins and Cancer

**DOI:** 10.3390/cancers15092421

**Published:** 2023-04-23

**Authors:** Mahmoud Toulany, Annette Lasham

**Affiliations:** 1 Division of Radiobiology & Molecular Environmental Research, Department of Radiation Oncology, University of Tuebingen, Roentgenweg 11, 72076 Tuebingen, Germany; 2 Department of Molecular Medicine and Pathology, School of Medical Sciences, The University of Auckland, Auckland 1142, New Zealand; 3 Maurice Wilkins Centre, The University of Auckland, Auckland 1010, New Zealand; 4 Te Aka Mātauranga Matepukupuku, Faculty of Medical and Health Sciences, The University of Auckland, Auckland 1142, New Zealand

Nine of the ten papers published in this Special Issue explore various aspects of the multifunctional protein Y-box binding protein-1 (YB-1) and its role in cancer. YB-1 is involved in many cellular processes, including cell proliferation, survival, and migration, and is known to be overexpressed in a wide range of cancers. These papers shed light on the molecular mechanisms underlying YB-1 function, its regulation, and its potential as a therapeutic target.

Morgenroth et al. investigated whether an autoimmune response develops against cancerous human YB-1 with post-translational protein modifications. They performed a systematic analysis of autoantibody formation directed against conformational and linear epitopes within the protein and detected autoantibodies against prokaryotic, but not eukaryotic full-length and cleaved human YB-1 protein fragments in both healthy volunteers and cancer patients. This study highlights the potential of using YB-1 autoantibodies as a diagnostic marker for cancer [1].

In the study carried out by Tiwari et al. the authors investigated the underlying signaling pathway involved in YB-1 phosphorylation in triple-negative breast cancer (TNBC) cells. They demonstrated that YB-1 is a key player in cell proliferation, clonogenic activity, and tumor growth of TNBC cells through the MAPK and PI3K pathways. They suggested that dual inhibition of these two pathways or the individual targeting of YB-1 may be an effective strategy to treat TNBC [2].

Mehta et al. found that YB-1 depletion in several cancer cell lines and in immortalized fibroblasts resulted in cytokinesis failure and consequent multinucleation. YB-1 was required for the completion of cytokinesis and orchestrating the spatio-temporal distribution of the microtubules, beta-actin, and the chromosome passenger complex (CPC) to define the cleavage plane. These findings suggest that YB-1 plays a critical role in cytokinesis and regulates cell division [3].

In the paper by Kosnopfel et al. the authors showed that YB-1 is a secreted factor that stimulates melanoma cell migration and invasion in a tumor progression stage-dependent manner. YB-1 can activate different signaling pathways in melanoma cells, leading to increased cell migration and invasion. This study highlights the potential of YB-1 as a therapeutic target for melanoma treatment [4].

Johnson et al. utilized an RNA-seq approach to investigate the effects of YB-1 knockdown on mesothelioma cell lines. They found that YB-1 knockdown regulates a set of genes enriched for regulators of mitosis, integrins, and extracellular matrix organization. Each cell line displayed a unique gene expression signature, suggesting that theloss of YB-1 affects a core set of genes in mesothelioma cells but also interacts with the genetic landscape of the cell [5].

The study by Shah et al. identified a crucial role for YB-1 in TNF-induced activation and nuclear translocation of NF-κB p65. They observed that YB-1-deficient cells were more prone to TNF-induced apoptotic cell death, suggesting that YB-1 plays a central role in promoting cell survival through NF-kB activation. These results identify a novel mechanism through which enhanced YB-1 expression may contribute to tumor development [6].

A second paper by Mehta et al. investigated the cellular localization of YB-1 during the cell cycle. They found that YB-1 enters the nucleus during the late G2/M phase and exits at the completion of mitosis, and that dephosphorylation of YB-1 at specific serine residues increased the accessibility of the nuclear localization signal, facilitating nuclear entry during late G2/M phase [7].

Sobočan et al. studied the communication between the PI3K/AKT/mTOR pathway and YB-1 in gynecological cancers. They found that women with increased levels of mTOR signaling pathway targets have a worse prognosis compared to those with normal mTOR levels. Targeting mTOR alone has led to unsatisfactory outcomes in gynecological cancer patients, highlighting the need for a more targeted approach [8].

The two review articles, “Cold-Shock Domains—Abundance, Structure, Properties, and Nucleic-Acid Binding” [9] and “Novel Insights into YB-1 Signaling and Cell Death Decisions” [10] provide valuable insights into the functions and mechanisms of two important proteins in biology. The cold-shock domain protein family and YB-1 play crucial roles in regulating transcription, translation, DNA damage repair, RNA splicing, and stress responses. The conserved structure and nucleic-acid binding properties of the cold-shock domain enable these proteins to associate with both DNA and RNA strands, providing a rationale for their diverse functions. Meanwhile, the role of YB-1 in activating NF-κB and signaling cell survival highlights its potential as a therapeutic target for inflammatory diseases and tumor therapy.

The two review articles by Heinemann and Roske [9], and Shah et al. [10], showcase the importance of understanding the structure, properties, and functions of the CSPs in biology. They serve as a reminder that even seemingly simple proteins such as those with the cold-shock domain can have complex biology, and that investigating their mechanisms can lead to significant insights into cellular processes. Furthermore, understanding the signaling pathways and mechanisms of CSPs such as YB-1 is critical in the development of effective therapeutic strategies for treating various diseases. Overall, these review articles provide valuable contributions to the field of CSP research and inspire further investigation into the intricacies of CSP function and regulation.

Overall, the ten papers in this Special Issue contribute to our understanding of the molecular mechanisms underlying CSPs in cancer progression and therapy resistance, and provide insights into potential therapeutic strategies for cancer treatment. In particular, these studies demonstrate the complex and context-dependent roles of YB-1 in cancer progression and cell survival. Targeting YB-1 may provide a novel approach to cancer therapy; however, more research is needed to understand its specific roles in different cancer types and contexts.
